# Testing the Effectiveness of 3D Film for Laboratory-Based Studies of Emotion

**DOI:** 10.1371/journal.pone.0105554

**Published:** 2014-08-29

**Authors:** Daniel L. Bride, Sheila E. Crowell, Brian R. Baucom, Erin A. Kaufman, Caitlin G. O'Connor, Chloe R. Skidmore, Mona Yaptangco

**Affiliations:** Department of Psychology, University of Utah, Salt Lake City, Utah, United States of America; National Cancer Institute, National Institutes of Health, United States of America

## Abstract

Research in psychology and affective neuroscience often relies on film as a standardized and reliable method for evoking emotion. However, clip validation is not undertaken regularly. This presents a challenge for research with adolescent and young adult samples who are exposed routinely to high-definition (HD) three-dimensional (3D) stimuli and may not respond to older, validated film clips. Studies with young people inform understanding of emotional development, dysregulated affect, and psychopathology, making it critical to assess whether technological advances improve the study of emotion. In the present study, we examine whether 3D film is more evocative than 2D using a tightly controlled within-subjects design. Participants (*n*  =  408) viewed clips during a concurrent psychophysiological assessment. Results indicate that both 2D and 3D technology are highly effective tools for emotion elicitation. However, 3D does not add incremental benefit over 2D, even when individual differences in anxiety, emotion dysregulation, and novelty seeking are considered.

## Introduction

Every human experience is imbued with emotion [1], [2]. Emotions influence the perception of stimuli and subsequent course of action [3]. Emotions can facilitate or hinder social connection, attachment, and survival [4]. The manner in which emotions are experienced and regulated can alter physical health, academic and work performance, social functioning, and well-being [5]. Because healthy emotional development is a central task of childhood, adolescence, and emerging adulthood, there has been a steady increase in lab-based studies of emotion with young samples [6]. However, the tools used in affective neuroscience research often lag behind the technological context of today's youth.

Emotion research is founded upon the assumption that affective responses can be elicited and measured reliably in the laboratory. Ideally, the elicitation technique will evoke a replicable response while approximating the intensity of real-world stimuli. However, many lab-based protocols preserve one of these qualities at the expense of the other—methods with high internal validity often sacrifice ecological validity, whereas those that seek to replicate prior emotional experiences can lack methodological rigor. The presentation of emotionally evocative film clips offers one solution to this paradox [7], [8]. Film clips offer high reliability while also evoking commonly-experienced emotions. A disadvantage of this method is that visual technology is updated continually but not validated regularly as a research tool. Researchers have yet to test whether technological improvements (e.g., sharper resolution, larger screen sizes, better color reproduction) and/or new media formats (e.g., 3D) enhance the utility of film for research.

In recent years, studios have produced and promoted more 3D films, a trend matched by the home electronics industry. The ubiquity of 3D stimuli raises important considerations for researchers. Three-dimensional presentation could improve ecological validity, since the increased visual realism of 3D may mimic real-life emotional stimuli more effectively than 2D. If so, 3D technology would be valuable to affective neuroscientists, offering the same standardization and convenience as 2D film with increased validity. Nonetheless, 3D equipment is costly and requires the use of specialized glasses. More importantly, the majority of films are not produced in 3D. These limitations may have prevented researchers from examining 3D film as an emotion elicitation technique.

Over the past two decades, visual technology has advanced and there has also been a rapid increase in affective neuroscience research [9]. Technological improvements have allowed researchers to assess autonomic (ANS) and central nervous system (CNS) substrates of emotional responding more easily and at a lower cost. In particular, the costs of assessing ANS responses have dropped substantially, allowing more researchers to enter the field. Moreover, there are unique advantages to assessing ANS responses via peripheral psychophysiological techniques. These approaches are rooted in a rich theoretical tradition of emotion research, and the CNS correlates of peripheral measures are increasingly well-delineated [10]. Psychophysiological research can be conducted with a wider variety of protocols and stimuli within a shorter timeframe than most CNS measures. Finally, ANS assessments are especially well-suited for studies with younger samples or for those who do not respond well to the more invasive approaches used to measure brain activity. Thus, autonomic measures are foundational to emotion research.

Electrodermal and cardiac measures are among the most commonly used indices of emotional responding. Electrodermal activity (EDA) is the product of eccrine sweat gland activation, typically measured on the thenar eminence of the non-dominant palm. Because the eccrine sweat glands are enervated almost exclusively by cholinergic fibers of the sympathetic nervous system (SNS), EDA is a reliable index of SNS responding to affective stimuli [11], [12]. The central pathways that control EDA have been outlined across several animal and human studies [13]–[15]. Briefly, EDA is correlated with brain activation across regions that assess the significance of stimuli (e.g., the ventromedial prefrontal cortex, right inferior parietal region, and the anterior cingulate). When the stimulus has an emotional valence, the amygdala and orbitofrontal cortex are also activated, typically producing a more robust electrodermal response. Because the eccrine glands are less responsive to thermal changes, EDA can be linked to psychological processes such as attention, arousal, and emotion—especially if the task is selected carefully and the experiment is well controlled [16], [17]. During rest, the number of electrodermal responses is typically low and the eccrine glands remain in a tonic state. Phasic EDA increases in response to challenging tasks (e.g., mental arithmetic) or strong emotions (e.g., rage or fear). Two common measures of eccrine sweat gland activity include: (1) phasic EDA, which is the number of non-specific electrodermal fluctuations during a task and (2) tonic period, which is the amount of time spent in a non-responsive state.

Three measures of cardiac physiology include heart rate (HR), cardiac pre-ejection period (PEP), and respiratory sinus arrhythmia (RSA). Of these, HR is the most widely reported, perhaps because HR can be interpreted easily and is correlated with other measures of physical and emotional health [18]. However, activity of the cardiovascular system is affected by both the sympathetic (SNS) and parasympathetic (PNS) branches of the ANS. Furthermore, the SNS and PNS can affect HR reciprocally, coactively, or independently [19]. Thus, isolating the effects of sympathetic and parasympathetic activity is important for interpreting and conceptualizing the association between psychological constructs and cardiovascular output.

Increases in heart rate driven by the sympathetic nervous system can be measured with PEP, which is the time interval between left ventricular depolarization and the ejection of blood into the aorta. Shortened PEP intervals reflect stronger sympathetic activation in response to stimuli [20]. As with all psychophysiological measures, the interpretation of PEP as an index of psychological states depends largely upon the stimulus conditions [16]. For example, most healthy participants show attenuated PEP to fearful stimuli [21] and in response to monetary reward [22]. In other words, shortened PEP would be expected under conditions that elicit active avoidance or active approach.

Parasympathetic influences on cardiac activity are assessed with RSA, a measure of heart rate variability mediated by the vagus nerve [23]. The vagus exerts an inhibitory effect on cardiac output. During rest and conditions of low demand, inhibitory vagal control of HR is high, allowing the person to remain in a flexible regulatory state. In response to stressful stimuli, however, vagal control is withdrawn and HR increases so that the individual can meet environmental challenges. Following removal of the acute stressor, vagal activity returns to a tonic inhibitory state, allowing HR to return to baseline and attentional resources to be shifted away from the stressor. Among young adult samples, high RSA is usually associated with better modulation of attention and self-regulatory capacity [24]. Low RSA and more pronounced decreases during stress are often observed in clinical samples and under stimulus conditions that provoke emotion dysregulation [23], [25].

Although physiological responses to emotion-inducing films have been examined previously [7], it is unclear whether 3D film produces a more robust physiological response. Furthermore, it is unknown whether some participants are more receptive to the 3D effect. Three-dimensional stimuli could affect all participants uniformly or individual differences could moderate physiological responding to 3D. For example, higher levels of anxiety, emotion dysregulation, or novelty seeking could influence the extent to which a person responds physiologically to 3D. Evidence indicates that anxious individuals tend to shift attention toward threatening stimuli, whereas less anxious individuals direct attention away from threat [26]. Thus, 3D stimuli could produce a more robust electrodermal response among those who score high on trait anxiety, especially for clips that should provoke a strong sympathetic response (e.g., fear or excitement).

Novelty seeking (NS) is a personality trait characterized by an immoderate approach to reward cues, avoidance of monotony, and the pursuit of excitement. These impulsive personality traits are associated with striatal dopamine (DA) activity, especially under conditions that elicit approach motivation or behavior [16], [27]. Peripherally, PEP is a good marker of striatal DA activity during conditions of reward [16]. Compared with reward protocols (e.g., monetary incentives), passive viewing tasks are less likely to reveal large individual differences in PEP reactivity. However, participants who score higher on NS may be even less reactive to 3D technology (i.e., longer PEP), given that 3D technology is widespread and may be less exciting for those who engage regularly in thrill-seeking behaviors. Similarly, there may be individual differences in PNS reactivity to 3D. Across several studies, attenuated RSA has been linked to psychological problems characterized by emotional and/or behavioral dyscontrol [23]. Thus, participants who score higher on measures of emotion dysregulation may show less parasympathetic regulation (i.e., lower RSA) in response to 3D film.

Following from this review, the present study was designed to test whether 3D film is a more powerful elicitation technique for laboratory studies of emotion and whether individual differences might moderate the response to 3D. We assessed this in a large sample of young adults using electrodermal and cardiac measures of reactivity as objective markers of the affective response. Because 3D stimuli offer enhanced visual realism, we hypothesized that 3D film would evoke a more robust physiological response than the same clip in 2D. Furthermore, we hypothesized that some people may be more responsive to 3D, even if differences did not emerge for the full sample. Therefore, we examined individual moderators of physiological responding to 3D stimuli, including trait anxiety, novelty seeking, and emotion dysregulation.

## Method

### Participants

The institutional review board at the University of Utah approved all study materials and procedures. Four hundred and eight adults between ages 18 and 64 participated (*M* = 24.4; *SD* = 7.6; 62.75% female). Nearly eighty-nine percent of participants identified their ethnicity as non-Hispanic (88.97%), 7.35% identified as Hispanic, and 15 did not report ethnicity. Almost eighty percent of the sample identified as White (79.66%), 9.8% identified as Asian, 1.23% as Black or African American, 1.23% as Native Hawaiian or Pacific Islander, 0.98% as American Indian or Alaska Native, and 29 participants did not report their race. Participants were recruited using two methods: a psychology department participant pool (*n* = 378) or fliers posted around campus and the surrounding community (*n* = 30). Those enrolling through the participant pool received research participation credit commensurate with the length of time required to complete the experiment. Those who responded to a flier were entered in a drawing for a gift card to an electronics store. The only inclusion criterion was that participants be over the age of 18. Participants were excluded who had a history of seizures or other neurological issues that could be affected by 3D technology. Because the university has many non-traditional students, we placed no upper limit on the age of participants.

### Film stimuli

Selection of film stimuli was constrained by the requirements of 3D viewing. Film clips were selected from among all titles available prior to the onset of the study on Blu-ray 3D disc—the industry standard for home-viewing of 3D media. All films likely to contain emotional content were assigned for viewing among a team of judges consisting of the authors and approximately 10 undergraduate research assistants. Undergraduates were consulted deliberately because they matched the age range of the intended sample [8]. After watching the films, judges nominated clips that elicited discrete target emotions—fear, sadness, amusement, and thrill/excitement. Candidate clips were then viewed by all raters and appropriate clips were chosen by consensus. Evaluation criteria included subjective intensity and clarity of the elicited emotion (i.e., the clip successfully elicited the target emotion more strongly than it elicited other emotions for a majority of judges), continuity (i.e., the clip was uninterrupted by irrelevant content or other emotions), context-independence (i.e., the emotional content was obvious without seeing other parts of the film or knowing the story), and appropriate length (greater than 2 min to facilitate sampling of physiological responding and less than 6 min to reduce participant fatigue and attentional decay). Time points and target emotions for the final selection of clips are as follows: *My Bloody Valentine* (1:01:00–1:06:00; fear), *Despicable Me* (36:10–40:10; amusement), *Tangled* (1:21:50–1:26:50; sadness), and *The Polar Express* (36:00–41:00; thrill/excitement).

### Procedure

Study procedures were completed in a comfortable, sound attenuated room. After obtaining written informed consent, an experimenter attached physiological electrodes to the participant and dimmed the room lights. The experimenter then instructed the participant to sit quietly during a 5 min resting baseline preceding the first film clip. After each film clip, the participant completed an on-screen questionnaire followed by a 90 s recovery/baseline period. During all baselines, large plain text was displayed on the screen to instruct participants to sit quietly but remain awake. Each study participant viewed a set of films clips in one presentation format (2D or 3D) followed by the same clips in the same order in the other format. The two clips were selected randomly from our pool of four clips, resulting in six possible pairings. The order in which the two clips were paired was also randomized, resulting in 12 possible unique configurations. Finally, the order of presentation format (2D or 3D first) was randomized, resulting in a total of 24 possible stimulus conditions. Each participant was assigned randomly to one of these 24 conditions. This counterbalanced design (grouping clips by presentation format and holding clip order constant across formats) allowed us to compare order effects as well as format effects.

The films were played from Blu-ray 3D discs on a Samsung BD-6900 Blu-ray 3D player and displayed on a Samsung UNC55C7000 55-inch 3D television using active shutter 3D glasses. The viewing room was designed to replicate the proportions of a typical movie theater but on a smaller scale. The room measured 112.5 inches by 104 inches and participants were seated approximately 6 feet from the 55-inch display.

### Measures

Each participant completed a series of self-report measures, which we included as potential moderators of the 2D vs. 3D effect. The *Temperament and Character Inventory-140* (TCI) [28] measures the proposed personality facets of Cloninger's biosocial model. We tested the NS subscale of the TCI as a moderator of PEP reactivity to 3D film. Novelty seeking is theorized to be associated with impulsivity and dopaminergic activity, which both have known associations with PEP [16]. The NS scale of the TCI has good internal consistency (α = .78) and a study re-examining the factor structure of the TCI found that this scale loaded on an independent factor [29].

The *State and Trait Anxiety Inventory-Form Y* (STAI-Y) [30] is a 40-item measure of both state and trait anxiety. We examined trait anxiety, as measured by the STAI-Y, as a moderator of EDA. Reliability for the trait subscale is excellent (α = .89) as is test-retest reliability (*ρ* = .88) [31].

The *Difficulties in Emotion Regulation Scale* (DERS) [32] is a widely used measure of emotion dysregulation. The DERS has high internal consistency (α = .93), good test-retest reliability (*ρ_I_* = .88), and adequate construct and predictive validity. We tested whether the DERS total scores moderated RSA reactivity to 3D film.

### Psychophysiology

We measured cardiac impedance, electrocardiography, and skin conductance using a MindWare Bionex 3711-08 chassis and MindWare Biolab 3.0.9 acquisition software (Mindware, Gahanna, OH, USA). We scored physiological data using MindWare EDA 3.0.9, HRV 3.0.10, and IMP 3.0.10 software suites (MindWare, Gahanna, OH, USA). Raw physiological data were scored in 30 s epochs to facilitate flexible analyses for a separate project. All statistical analyses for the current project were conducted on change scores, a common measure of physiological reactivity. Change scores were created by subtracting each 30 s epoch from the average score across the two baseline epochs immediately preceding the clip. Change scores calculated in relation to the preceding baseline are preferred over those calculated from an initial baseline period. Whereas the former approach isolates physiological changes evoked by the current stimulus from residual effects of the prior stimulus, the latter may confound the effects of the current stimulus with lingering effects from any interceding stimuli. Change scores for adjacent 30 s epochs were averaged to make 1 min epochs, a standard length for cardiovascular psychophysiology measurements [10]. These 1 min average change scores are mathematically equivalent to change scores created by scoring the same data in 1 min epochs.

Physiological data were scored in a three step process by trained research assistants and the first author. First, we obtained physiological scores via computer algorithm using the appropriate MindWare physiology scoring software. Next, we inspected physiological signal data visually for each 30 second epoch to confirm correct placement of physiological markers (e.g., QRS points, troughs and peaks of EDA responses), which determine physiological scores. Misplaced markers (e.g., due to signal distortion) were corrected manually when correct placement was visually apparent or flagged for further review. Finally, all flagged data were reviewed by the first author, who consulted corresponding video of the experimental session to remove artifacts caused by participant movement (e.g., sneezing, fidgeting) and substitute missing data codes when deriving a score was not possible.

Prior to conducting statistical analyses, physiological data were screened for errors by the first author in two steps. First, scored physiological signal data for every third participant were inspected visually to ensure research assistants' conformity to standardized scoring procedures. Second, all physiological scores were entered in a spreadsheet, grouped by film clip and type of physiology. We confirmed that all resting psychophysiological scores fell within a typical range for each biological measure by checking baseline scores (the average across the final two 30 s epochs of resting data). For EDA, the number of responses ranged from 0–8.5 (*M* = 1.25, *SD* = 1.6). Tonic period ranged from 9.6–30.0 s (*M* = 25.6, *SD* = 4.7). The range for HR was 46.8–115.8 (*M* = 73.3, *SD* = 11.4). Range for RSA was .90–9.77 (*M* = 6.1, *SD* = 1.3) and PEP was 70.43–155.5 (*M* = 121.5, *SD* = 11.6). Highlighting algorithms were applied to identify values that approached or exceeded the putative upper or lower limits of each type of physiological score, scores that were more than one standard deviation from the sample mean for each type of physiology, and within-participant epoch-to-epoch changes in physiology scores that were larger than expected. Suspicious scores were reviewed by the first author and corrected when necessary using the scoring procedures outlined above. No outliers were identified once the cleaning process had been completed.

## Results

### Statistical Approach

**Figure 1:**
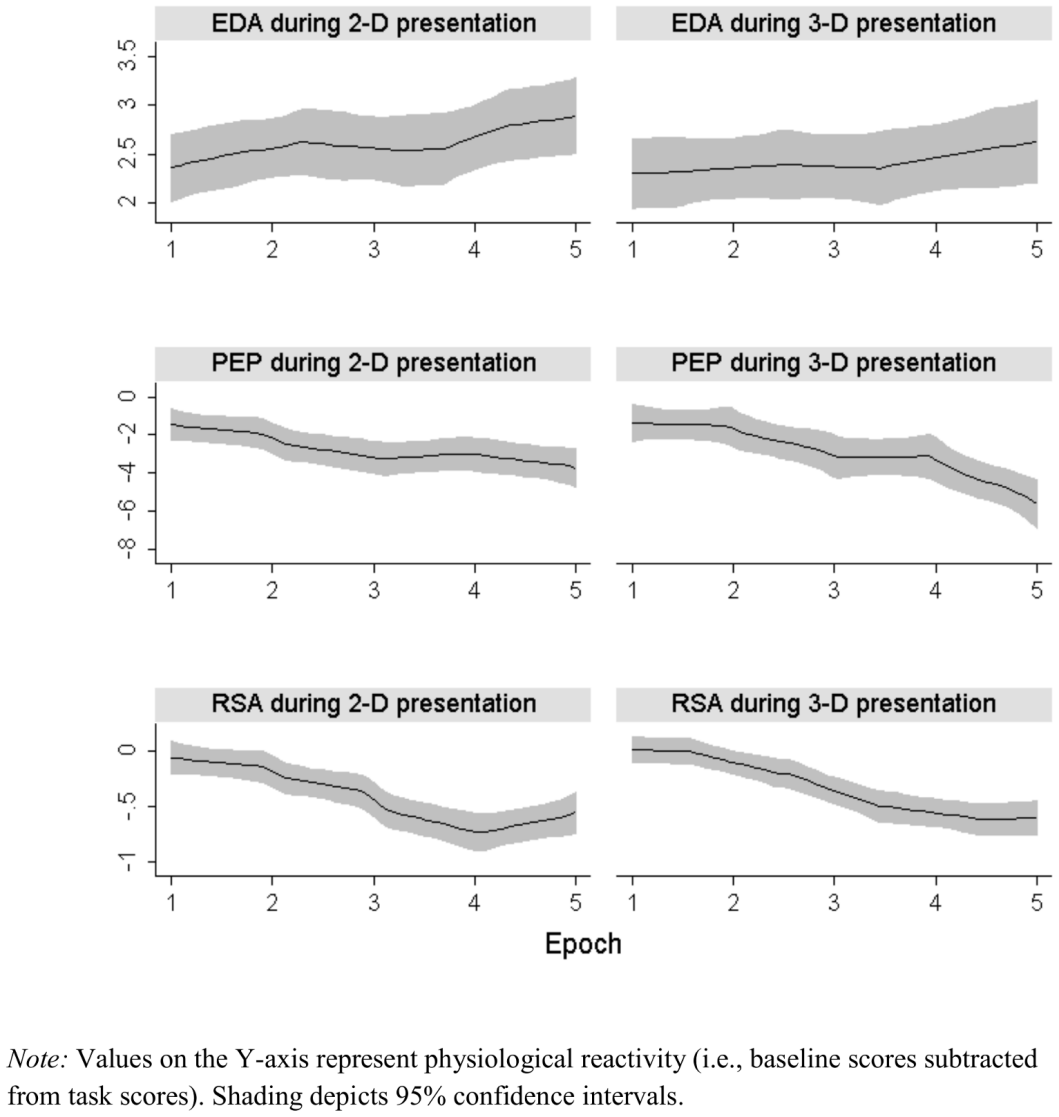
Local polynomial smoothed plots of EDA, PEP, and RSA in 2D and 3D during the first presentation of *My Bloody Valentine*. Values on the Y-axis represent physiological reactivity (i.e., baseline scores subtracted from task scores). Shading depicts 95% confidence intervals.

We tested hypotheses with a series of multilevel models using the Hierarchical Linear and Nonlinear Modeling program [33]. In step one, initial models were developed to characterize change over time in each physiological measure. Given the complexity of our hypotheses, our analytic strategy was to create models that allowed for subtle differences in change over time while also being as parsimonious as possible. Fixed and random effects for linear, quadratic, and cubic time parameters were evaluated for all physiological measures using procedures recommended by Singer and Willett [34]. A higher order parameter of time was retained only if the fixed or random effect for that model was significant and resulted in a substantial decrease in the deviance statistic. Additionally, plots of temporal variation in each form of physiological reactivity were visually inspected for evidence that retaining higher order polynomials appeared warranted. As one example, Figure 1 displays the local polynomial smoothed plots used to inspect temporal variation in EDA, PEP, and RSA during My Bloody Valentine. In step two, differences between physiological reactivity to 2D and 3D film clips and due to order of presentation were tested by introducing two dummy coded variables (for 2D vs. 3D: 0 = 2D, 1 = 3D; for order of presentation: 0 =  first presentation, 1 =  second presentation) as well as interactions between these dummy coded variables and all time parameters at Level-1. In step three, individual difference moderators were examined by adding grand-mean centered moderator variables at Level-2. Given established sex differences on physiological measures [35], sex was included at Level-2 as an effect-coded covariate (−.5 =  female, .5 =  male) for all lower level parameters in all models. All models were also run including age as a covariate. Because the distribution of age was skewed, separate models were run where age was scored on the original metric as well as where age was transformed using an inverse transformation and dichotomized into individuals above and below the age of 40 (0  =  under 40, 1  =  over 40). Age effects were non-significant in 17 of the 20 models, and age effects involving interactions with 2D vs. 3D presentation were non-significant in 19 of the 20 models. In the one significant interaction between age and 2D vs. 3D presentation, older adults had an increase in the amount of time spent in a non-responsive electrodermal state (i.e., a longer tonic period; TON) during the 3D presentation of *Polar Express* but not during the 2D presentation. In other words, older adults habituated to the 3D presentation more quickly. Because age appears to have a very minor effect at most, it was not included as a covariate in additional analyses in order to reduce model complexity. Time was centered so that the intercept indexes physiological reactivity during the first epoch. The following equation describes the complete two-level model used to evaluate moderation of the linear effect of time:

Level-1:
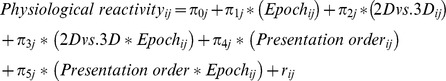



Level-2:
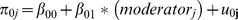



where *i* indexes epoch and *j* indexes individuals. In order to allow for differences in the shape of change over time between film clips and physiological measures, separate models were run for each physiological measure in each film clip. A family-wise Bonferroni correction was applied to analyses for each film clip to correct for the possibility of Type I error arising from multiple comparisons in steps 2 and 3; alpha was therefore set at .01 for these analyses.

### Testing for 2D-3D Differences

Table S1 displays results of all initial models. The significant effects of time in each model demonstrate reactivity across each physiological measure to each film clip. In other words, participants responded to the film clips across all physiological measures, as would be expected for emotionally evocative stimuli. Table S2 displays results of models testing differences in reactivity to 2D vs 3D clips and due to order of presentation. Taken as a whole, very few significant differences emerged in physiological reactivity to 3D film clips relative to 2D film clips; there were no significant differences in 19 of the 20 models. The only significant difference that emerged was that participants had more electrodermal responses during the 3D version of *The Polar Express* relative to the 2D version. In contrast to these scant differences, significant presentation order effects emerged in 14 of the 20 models. These effects indicated a consistently stronger physiological response to the first presentation relative to the second presentation. Finally, two significant gender differences emerged: (1) men were less electrodermally responsive during the first presentation of *Polar Express* (i.e., greater tonic period) than women and (2) women had more EDA responses during the first presentation of *My Bloody Valentine* than men. Women's and men's EDA responses during the second presentation of *My Bloody Valentine* were not different from one another. In other words, women were more reactive to stimuli eliciting fear and thrill/excitement. Table S3 displays results of individual difference moderator models. None of the effects for any of the other moderators emerged as significant predictors of the 3D response.

## Discussion

Three-dimensional film is increasingly available in theaters and is becoming a popular feature for home audio/video systems. Additionally, most films produced in 3D are targeted to children, adolescents, and young adults. If the popularity of this viewing format continues, future generations may be exposed to 3D technology as frequently as 2D content. One could argue that 3D film produces a more vivid, lifelike experience. Indeed, marketing strategists promote 3D as more thrilling and intense, as though the viewer were experiencing the setting firsthand. This begs the question—does 3D film elicit stronger emotional reactions than the same content in 2D?

The purpose of this study was to probe this question using a carefully controlled experimental approach. We accomplished this by manipulating the presentation format and measuring psychophysiological reactivity (i.e., physiological change from baseline). The decision to use psychophysiological outcomes and film-based stimuli follows from the growing literature that employs these techniques, including basic and clinical studies of emotion [36], [37]. Although other stimuli (e.g., virtual reality) and outcome measures (e.g., BOLD signal change) are alternatives, many emotion researchers prefer techniques that are less invasive and more cost effective than these approaches.

Importantly, the films used in this study evoked patterns of psychophysiological reactivity consistent with the scenes presented. As illustrated in Figure 1, the fear-inducing clip (*My Bloody Valentine*) produced a robust sympathetic response, which is evidenced by increased EDA and shortened PEP. Participants also exhibited attenuated RSA during this clip, consistent with theories that parasympathetic withdrawal may mark emotion dysregulation [16]. The responses analyzed in this paper and graphed in Figure 1 show changes in physiological activity from baseline (i.e., reactivity). This is a conservative approach to examining the affective response because reactivity scores account for baseline physiological differences. Based on these findings, *any of these clips could be useful for future studies of emotion*. However, in nearly all cases 3D technology did not enhance the viewing experience.

Among the four film clips and five physiological measures tested, we observed only one difference between 2D and 3D formats—the number of electrodermal responses during *The Polar Express*, which was intended to elicit a response of thrill/excitement. Although a single finding should be interpreted with caution, there are many reasons why *The Polar Express 3D* should evoke a stronger psychophysiological response. This clip was one of the highest quality 3D films and included a larger variety and number of 3D effects. Moreover, the 3D content in this clip is more sustained—lasting almost the full duration of the clip as opposed to the briefer effects used in the other films. Further research should examine whether such differences in type, quality, intensity, and duration of 3D content produce more robust physiological responses.

Our results are bolstered by the finding that individual differences in emotion dysregulation, anxiety, and NS did not moderate the psychophysiological response to 3D. Although we hypothesized that some people would be more responsive to 3D stimuli, individual differences did not account for the lack of 2D vs. 3D effect. Interestingly, emotion dysregulation did predict physiological reactivity *during both 2D and 3D* presentations of *Polar Express*. As would be expected, greater emotion dysregulation was associated with more pronounced RSA decreases during the clip. This suggests that *The Polar Express* clip may be especially distressing for participants who report greater difficulties with emotion dysregulation, which is consistent with the existing theoretical and empirical literature [25].

These results should be encouraging for researchers who lack the resources to incorporate 3D technology into their laboratory. Nonetheless, future studies should seek to replicate these findings. Our sample was comprised primarily of emerging adults, a group likely to be accustomed to and expect greater visual realism. Thus, the results may not generalize to younger or older participants, for whom the novelty of 3D content could produce a greater effect. Future research should also test whether more sustained 3D content produces better results. We suspect that the techniques employed in *The Polar Express 3D* may be responsible for the finding of greater EDA during this clip. However, it is possible that this was a chance finding, even with the family-wise Bonferroni correction used to control for Type 1 error. Finally, future research could also compare these films to previously-validated clips [7]. All of the films used in this study were produced in high-definition format and shown on a TV where the ratio of screen to room size approximated the movie theater experience. Thus, although there was no 2D–3D difference, any of these clips may be more evocative than those from older films of lower visual quality.

One need not look to affective neuroscience research to realize the importance of understanding emotion. The portrait of a human life is painted with emotion, which colors memory and guides behavior [2]. When attempting to replicate the rich emotional experience of everyday life, laboratory research is in its infancy. Still, no matter how banal, the laboratory setting offers advantages for carefully controlled research on emotion, making it critical that researchers employ the best available methods. Testing the benefit of 3D over 2D is just one step toward a more realistic laboratory-based emotional experience—and the contrast may not have been dramatic enough. Indeed, our results suggest that participants respond to the content and novelty of film more strongly than to the visual technology. Future research can build upon this finding to test newer, and more realistic visual stimuli as a means of improving the science of emotion elicitation.

## Supporting Information

Table S1 **Results of initial multilevel models.** Robust standard errors are reported. ^*^ p<.05, ^**^ p<.01, ^***^ p<.001.
(DOCX)

Table S2 **Results of multilevel models testing differences in physiological reactivity to 2D and 3D presentations.** Robust standard errors are reported. ^*^ p<.05, ^**^ p<.01, ^***^ p<.001.
(DOCX)

Table S3 **Results of Analyses Testing whether Individual Differences Moderate the 3D Response.** Robust standard errors are reported. ^*^ p<.05, ^**^ p<.01, ^***^ p<.001.
(DOCX)

Dataset S1 **Physiology data.**
(CSV)

Dataset S2 **Self-report measures.**
(CSV)

Codebook S1 **Physiology codebook.**
(CSV)

Codebook S2 **Self-report codebook.**
(CSV)
