# Supplementary material for: Testing the Effectiveness of 3D Film for Laboratory-Based Studies of Emotion
Source: PLoS One. 2014 Aug 29;9(8):e105554. doi: 10.1371/journal.pone.0105554 (PMC4149373; doi:10.1371/journal.pone.0105554)
Supplement: Table S1 — Results of initial multilevel models. Robust standard errors are reported. * p<.05, ** p<.01, *** p<.001. (DOCX) [file pone.0105554.s001.docx]

Table S1

*Results of initial multilevel models*

| Variable | | *B* | | *SE B* |
| --- | --- | --- | --- | --- |
|  | Despicable Me | |  | |
| EDA | |  | |  |
| Intercept, *β_00_* | | 1.17^***^ | | 0.14 |
| Gender, *β_01_* | | 0.24 | | 0.27 |
| Epoch, *β_10_* | | -0.65^***^ | | 0.09 |
| Gender, *β_11_* | | 0.11 | | 0.17 |
| Epoch^2^, *β_20_* | | 0.17^***^ | | 0.03 |
| Gender, *β_21_* | | -0.02 | | 0.05 |
| Epoch^3^, *β_30_* | | --- | | --- |
| Gender, *β_31_* | | --- | | --- |
| 2-D vs. 3-D, *β_40_* | | -0.23 | | 0.12 |
| Gender, *β_41_* | | 0.12 | | 0.25 |
| Presentation order, *β_50_* | | -0.80^***^ | | 0.12 |
| Gender, *β_51_* | | -0.18 | | 0.25 |
| Heart rate | |  | |  |
| Intercept, *β_00_* | | -1.37^***^ | | 0.34 |
| Gender, *β_01_* | | -0.66 | | 0.69 |
| Epoch, *β_10_* | | -0.82^***^ | | 0.22 |
| Gender, *β_11_* | | 0.23 | | 0.43 |
| Epoch^2^, *β_20_* | | 0.30^***^ | | 0.06 |
| Gender, *β_21_* | | -0.13 | | 0.12 |
| Epoch^3^, *β_30_* | | --- | | --- |
| Gender, *β_21_* | | --- | | --- |
| 2-D vs. 3-D, *β_40_* | | -0.03 | | 0.32 |
| Gender, *β_41_* | | -0.25 | | 0.64 |
| Presentation order, *β_50_* | | 1.28^***^ | | 0.32 |
| Gender, *β_51_* | | 0.62 | | 0.64 |
| PEP | |  | |  |
| Intercept, *β_00_* | | -0.95^*^ | | 0.38 |
| Gender, *β_01_* | | -0.75 | | 0.75 |
| Epoch, *β_10_* | | 0.38^***^ | | 0.09 |
| Gender, *β_11_* | | -0.19 | | 0.18 |
| Epoch^2^, *β_20_* | | --- | | --- |
| Gender, *β_21_* | | --- | | --- |
| Epoch^3^, *β_30_* | | --- | | --- |
| Gender, *β_31_* | | --- | | --- |
| 2-D vs. 3-D, *β_40_* | | 0.40 | | 0.48 |
| Gender, *β_41_* | | 0.19 | | 0.96 |
| Presentation order, *β_50_* | | -0.48 | | 0.48 |
| Gender, *β_51_* | | 0.76 | | 0.96 |
| RSA | |  | |  |
| Intercept, *β_00_* | | 0.05 | | 0.07 |
| Gender, *β_01_* | | 0.14 | | 0.14 |
| Epoch, *β_10_* | | -0.06^***^ | | 0.01 |
| Gender, *β_11_* | | 0.01 | | 0.03 |
| Epoch^2^, *β_20_* | | --- | | --- |
| Gender, *β_21_* | | --- | | --- |
| Epoch^3^, *β_30_* | | --- | | --- |
| Gender, *β_31_* | | --- | | --- |
| 2-D vs. 3-D, *β_40_* | | -0.07 | | 0.07 |
| Gender, *β_41_* | | 0.05 | | 0.13 |
| Presentation order, *β_50_* | | -0.09 | | 0.07 |
| Gender, *β_51_* | | -0.04 | | 0.13 |
| Tonic period | |  | |  |
| Intercept, *β_00_* | | -3.38^***^ | | 0.50 |
| Gender, *β_01_* | | -0.63 | | 1.00 |
| Epoch, *β_10_* | | 1.98^***^ | | 0.31 |
| Gender, *β_11_* | | -1.08 | | 0.62 |
| Epoch^2^, *β_20_* | | -0.57^***^ | | 0.10 |
| Gender, *β_21_* | | 0.24 | | 0.19 |
| Epoch^3^, *β_30_* | | --- | | --- |
| Gender, *β_31_* | | --- | | --- |
| 2-D vs. 3-D, *β_40_* | | 0.02 | | 0.45 |
| Gender, *β_41_* | | 0.27 | | 0.91 |
| Presentation order, *β_50_* | | 2.32^***^ | | 0.45 |
| Gender, *β_51_* | | -0.01 | | 0.91 |
|  | My Bloody Valentine | |  | |
| EDA | |  | |  |
| Intercept, *β_00_* | | 2.57^***^ | | 0.20 |
| Gender, *β_01_* | | -0.61 | | 0.39 |
| Epoch, *β_10_* | | -0.27^*^ | | 0.11 |
| Gender, *β_11_* | | 0.72^**^ | | 0.23 |
| Epoch^2^, *β_20_* | | 0.07^***^ | | 0.02 |
| Gender, *β_21_* | | -0.13^**^ | | 0.05 |
| Epoch^3^, *β_30_* | | --- | | --- |
| Gender, *β_31_* | | --- | | --- |
| 2-D vs. 3-D, *β_40_* | | -0.02 | | 0.18 |
| Gender, *β_41_* | | -0.03 | | 0.36 |
| Presentation order, *β_50_* | | -1.46^***^ | | 0.18 |
| Gender, *β_51_* | | 0.46 | | 0.36 |
| Heart rate | |  | |  |
| Intercept, *β_00_* | | 0.40 | | 0.49 |
| Gender, *β_01_* | | -0.76 | | 0.98 |
| Epoch, *β_10_* | | 0.82^***^ | | 0.11 |
| Gender, *β_11_* | | 0.25 | | 0.22 |
| Epoch^2^, *β_20_* | | --- | | --- |
| Gender, *β_21_* | | --- | | --- |
| Epoch^3^, *β_30_* | | --- | | --- |
| Gender, *β_31_* | | --- | | --- |
| 2-D vs. 3-D, *β_40_* | | 0.30 | | 0.37 |
| Gender, *β_41_* | | -0.29 | | 0.75 |
| Presentation order, *β_50_* | | -1.25^***^ | | 0.37 |
| Gender, *β_51_* | | -0.09 | | 0.75 |
| PEP | |  | |  |
| Intercept, *β_00_* | | -2.54^***^ | | 0.49 |
| Gender, *β_01_* | | 1.34 | | 0.98 |
| Epoch, *β_10_* | | -0.39^***^ | | 0.11 |
| Gender, *β_11_* | | -0.04 | | 0.22 |
| Epoch^2^, *β_20_* | | --- | | --- |
| Gender, *β_21_* | | --- | | --- |
| Epoch^3^, *β_30_* | | --- | | --- |
| Gender, *β_31_* | | --- | | --- |
| 2-D vs. 3-D, *β_40_* | | 0.02 | | 0.45 |
| Gender, *β_41_* | | -0.27 | | 0.91 |
| Presentation order, *β_50_* | | 1.91^***^ | | 0.45 |
| Gender, *β_51_* | | -0.85 | | 0.91 |
| RSA | |  | |  |
| Intercept, *β_00_* | | 0.01 | | 0.07 |
| Gender, *β_01_* | | -0.14 | | 0.15 |
| Epoch, *β_10_* | | -0.34^***^ | | 0.04 |
| Gender, *β_11_* | | 0.02 | | 0.08 |
| Epoch^2^, *β_20_* | | 0.04^***^ | | 0.01 |
| Gender, *β_21_* | | -0.01 | | 0.02 |
| Epoch^3^, *β_30_* | | --- | | --- |
| Gender, *β_31_* | | --- | | --- |
| 2-D vs. 3-D, *β_40_* | | 0.06 | | 0.08 |
| Gender, *β_41_* | | 0.17 | | 0.15 |
| Presentation order, *β_50_* | | 0.11 | | 0.08 |
| Gender, *β_51_* | | 0.06 | | 0.15 |
| Tonic period | |  | |  |
| Intercept, *β_00_* | | -6.49^***^ | | 0.51 |
| Gender, *β_01_* | | -0.61 | | 1.02 |
| Epoch, *β_10_* | | 1.41^***^ | | 0.30 |
| Gender, *β_11_* | | -0.71 | | 0.60 |
| Epoch^2^, *β_20_* | | -0.37^***^ | | 0.07 |
| Gender, *β_21_* | | 0.09 | | 0.13 |
| Epoch^3^, *β_30_* | | --- | | --- |
| Gender, *β_31_* | | --- | | --- |
| 2-D vs. 3-D, *β_40_* | | -0.11 | | 0.53 |
| Gender, *β_41_* | | 0.49 | | 1.06 |
| Presentation order, *β_50_* | | 3.22^***^ | | 0.53 |
| Gender, *β_51_* | | -0.35 | | 1.06 |
|  | Polar Express | |  | |
| EDA | |  | |  |
| Intercept, *β_00_* | | 2.17^***^ | | 0.16 |
| Gender, *β_01_* | | 0.14 | | 0.33 |
| Epoch, *β_10_* | | -1.52^***^ | | 0.10 |
| Gender, *β_11_* | | 0.26 | | 0.21 |
| Epoch^2^, *β_20_* | | 0.28^***^ | | 0.02 |
| Gender, *β_21_* | | -0.05 | | 0.04 |
| Epoch^3^, *β_30_* | | --- | | --- |
| Gender, *β_31_* | | --- | | --- |
| 2-D vs. 3-D, *β_40_* | | 0.27 | | 0.16 |
| Gender, *β_41_* | | -0.29 | | 0.33 |
| Presentation order, *β_50_* | | -0.94^***^ | | 0.16 |
| Gender, *β_51_* | | 0.14 | | 0.33 |
| Heart rate | |  | |  |
| Intercept, *β_00_* | | -1.42^***^ | | 0.38 |
| Gender, *β_01_* | | 0.96 | | 0.75 |
| Epoch, *β_10_* | | -4.54^***^ | | 0.30 |
| Gender, *β_11_* | | -0.20 | | 0.61 |
| Epoch^2^, *β_20_* | | 2.84^***^ | | 0.18 |
| Gender, *β_21_* | | 0.24 | | 0.36 |
| Epoch^3^, *β_30_* | | -0.42^***^ | | 0.03 |
| Gender, *β_31_* | | -0.04 | | 0.06 |
| 2-D vs. 3-D, *β_40_* | | 0.29 | | 0.27 |
| Gender, *β_41_* | | -1.16^*^ | | 0.55 |
| Presentation order, *β_50_* | | 1.06^***^ | | 0.27 |
| Gender, *β_51_* | | 0.22 | | 0.55 |
| PEP | |  | |  |
| Intercept, *β_00_* | | -1.53^**^ | | 0.47 |
| Gender, *β_01_* | | 1.36 | | 0.93 |
| Epoch, *β_10_* | | 0.94^***^ | | 0.29 |
| Gender, *β_11_* | | 0.18 | | 0.59 |
| Epoch^2^, *β_20_* | | -0.11^*^ | | 0.05 |
| Gender, *β_21_* | | -0.06 | | 0.10 |
| Epoch^3^, *β_30_* | | --- | | --- |
| Gender, *β_31_* | | --- | | --- |
| 2-D vs. 3-D, *β_40_* | | 0.11 | | 0.41 |
| Gender, *β_41_* | | -0.06 | | 0.81 |
| Presentation order, *β_50_* | | -0.54 | | 0.41 |
| Gender, *β_51_* | | -0.15 | | 0.81 |
| RSA | |  | |  |
| Intercept, *β_00_* | | -0.25^**^ | | 0.09 |
| Gender, *β_01_* | | -0.26 | | 0.18 |
| Epoch, *β_10_* | | 0.18^***^ | | 0.04 |
| Gender, *β_11_* | | 0.04 | | 0.09 |
| Epoch^2^, *β_20_* | | -0.05^***^ | | 0.01 |
| Gender, *β_21_* | | -0.01 | | 0.01 |
| Epoch^3^, *β_30_* | | --- | | --- |
| Gender, *β_31_* | | --- | | --- |
| 2-D vs. 3-D, *β_40_* | | 0.10 | | 0.07 |
| Gender, *β_41_* | | -0.17 | | 0.14 |
| Presentation order, *β_50_* | | -0.18^*^ | | 0.07 |
| Gender, *β_51_* | | 0.43^**^ | | 0.14 |
| Tonic period | |  | |  |
| Intercept, *β_00_* | | -3.72^***^ | | 0.40 |
| Gender, *β_01_* | | -2.36^**^ | | 0.79 |
| Epoch, *β_10_* | | 0.98^***^ | | 0.07 |
| Gender, *β_11_* | | -0.01 | | 0.13 |
| Epoch^2^, *β_20_* | | --- | | --- |
| Gender, *β_21_* | | --- | | --- |
| Epoch^3^, *β_30_* | | --- | | --- |
| Gender, *β_31_* | | --- | | --- |
| 2-D vs. 3-D, *β_40_* | | -0.64 | | 0.38 |
| Gender, *β_41_* | | 1.85^*^ | | 0.77 |
| Presentation order, *β_50_* | | 1.68^***^ | | 0.38 |
| Gender, *β_51_* | | 0.17 | | 0.77 |
|  | Tangled | |  | |
| EDA | |  | |  |
| Intercept, *β_00_* | | 1.96^***^ | | 0.18 |
| Gender, *β_01_* | | 0.18 | | 0.35 |
| Epoch, *β_10_* | | -2.27^***^ | | 0.16 |
| Gender, *β_11_* | | -0.04 | | 0.33 |
| Epoch^2^, *β_20_* | | 1.24^***^ | | 0.10 |
| Gender, *β_21_* | | 0.00 | | 0.21 |
| Epoch^3^, *β_30_* | | -0.19^***^ | | 0.02 |
| Gender, *β_31_* | | 0.00 | | 0.03 |
| 2-D vs. 3-D, *β_40_* | | 0.12 | | 0.15 |
| Gender, *β_41_* | | 0.06 | | 0.30 |
| Presentation order, *β_50_* | | -0.94^***^ | | 0.15 |
| Gender, *β_51_* | | -0.02 | | 0.30 |
| Heart rate | |  | |  |
| Intercept, *β_00_* | | -1.50^***^ | | 0.39 |
| Gender, *β_01_* | | -0.01 | | 0.79 |
| Epoch, *β_10_* | | -1.99^***^ | | 0.28 |
| Gender, *β_11_* | | -0.11 | | 0.56 |
| Epoch^2^, *β_20_* | | 1.32^***^ | | 0.17 |
| Gender, *β_21_* | | 0.04 | | 0.35 |
| Epoch^3^, *β_30_* | | -0.22^***^ | | 0.03 |
| Gender, *β_31_* | | 0.00 | | 0.06 |
| 2-D vs. 3-D, *β_40_* | | 0.22 | | 0.37 |
| Gender, *β_41_* | | 0.47 | | 0.75 |
| Presentation order, *β_50_* | | 0.99^**^ | | 0.37 |
| Gender, *β_51_* | | -0.46 | | 0.74 |
| PEP | |  | |  |
| Intercept, *β_00_* | | 0.27 | | 0.52 |
| Gender, *β_01_* | | -0.46 | | 1.05 |
| Epoch, *β_10_* | | 1.20^**^ | | 0.43 |
| Gender, *β_11_* | | 1.13 | | 0.85 |
| Epoch^2^, *β_20_* | | -0.70^**^ | | 0.27 |
| Gender, *β_21_* | | -1.02 | | 0.05 |
| Epoch^3^, *β_30_* | | 0.12^**^ | | 0.04 |
| Gender, *β_31_* | | 0.18^*^ | | 0.09 |
| 2-D vs. 3-D, *β_40_* | | -0.74 | | 0.55 |
| Gender, *β_41_* | | -0.82 | | 1.09 |
| Presentation order, *β_50_* | | -0.35 | | 0.55 |
| Gender, *β_51_* | | 2.18^*^ | | 1.09 |
| RSA | |  | |  |
| Intercept, *β_00_* | | -0.14^*^ | | 0.07 |
| Gender, *β_01_* | | 0.11 | | 0.14 |
| Epoch, *β_10_* | | -0.21^***^ | | 0.03 |
| Gender, *β_21_* | | 0.05 | | 0.07 |
| Epoch^2^, *β_20_* | | 0.06^***^ | | 0.01 |
| Gender, *β_21_* | | -0.02 | | 0.02 |
| Epoch^3^, *β_30_* | | --- | | --- |
| Gender, *β_31_* | | --- | | --- |
| 2-D vs. 3-D, *β_40_* | | 0.07 | | 0.08 |
| Gender, *β_41_* | | -0.03 | | 0.15 |
| Presentation order, *β_50_* | | -0.08 | | 0.08 |
| Gender, *β_51_* | | 0.06 | | 0.15 |
| Tonic period | |  | |  |
| Intercept, *β_00_* | | -5.60^***^ | | 0.51 |
| Gender, *β_01_* | | 0.06 | | 1.01 |
| Epoch, *β_10_* | | 6.18^***^ | | 0.50 |
| Gender, *β_11_* | | 1.26 | | 0.99 |
| Epoch^2^, *β_20_* | | -3.56^***^ | | 0.30 |
| Gender, *β_21_* | | -0.78 | | 0.61 |
| Epoch^3^, *β_30_* | | 0.56^***^ | | 0.05 |
| Gender, *β_31_* | | 0.13 | | 0.10 |
| 2-D vs. 3-D, *β_40_* | | 0.41 | | 0.44 |
| Gender, *β_41_* | | -1.00 | | 0.87 |
| Presentation order, *β_50_* | | 2.25^***^ | | 0.44 |
| Gender, *β_51_* | | -1.17 | | 0.87 |

*Note:* Robust standard errors are reported

^*^ p < .05, ^**^ p < .01, ^***^ p < .001
