# Supplementary material for: Testing the Effectiveness of 3D Film for Laboratory-Based Studies of Emotion
Source: PLoS One. 2014 Aug 29;9(8):e105554. doi: 10.1371/journal.pone.0105554 (PMC4149373; doi:10.1371/journal.pone.0105554)
Supplement: Table S2 — Results of multilevel models testing differences in physiological reactivity to 2D and 3D presentations. Robust standard errors are reported. * p<.05, ** p<.01, *** p<.001. (DOCX) [file pone.0105554.s002.docx]

Table S2

*Results of multilevel models testing differences in physiological reactivity to 2D and 3D presentations*

| Variable | | *B* | | *SE B* |
| --- | --- | --- | --- | --- |
|  | Despicable Me | |  | |
| EDA | |  | |  |
| 2-D vs. 3-D, *β_40_* | | -0.04 | | 0.16 |
| Gender, *β_41_* | | 0.13 | | 0.31 |
| Epoch X 2-D vs. 3-D, *β_50_* | | -0.31 | | 0.16 |
| Gender, *β_51_* | | -0.24 | | 0.33 |
| Epoch^2^ X 2-D vs. 3-D, *β_60_* | | 0.09 | | 0.05 |
| Gender, *β_61_* | | 0.10 | | 0.10 |
| Epoch^3^ X 2-D vs. 3-D, *β_70_* | | --- | | --- |
| Gender, *β_71_* | | --- | | --- |
| Heart rate | |  | |  |
| 2-D vs. 3-D, *β_40_* | | -0.10 | | 0.38 |
| Gender, *β_41_* | | -0.66 | | 0.76 |
| Epoch X 2-D vs. 3-D, *β_50_* | | -0.11 | | 0.37 |
| Gender, *β_51_* | | 0.25 | | 0.74 |
| Epoch^2^ X 2-D vs. 3-D, *β_60_* | | 0.07 | | 0.11 |
| Gender, *β_61_* | | -0.02 | | 0.22 |
| Epoch^3^ X 2-D vs. 3-D, *β_70_* | | --- | | --- |
| Gender, *β_71_* | | --- | | --- |
| PEP | |  | |  |
| 2-D vs. 3-D, *β_40_* | | 0.40 | | 0.47 |
| Gender, *β_41_* | | 0.82 | | 0.94 |
| Epoch X 2-D vs. 3-D, *β_50_* | | 0.00 | | 0.17 |
| Gender, *β_51_* | | -0.42 | | 0.33 |
| Epoch^2^ X 2-D vs. 3-D, *β_60_* | | --- | | --- |
| Gender, *β_61_* | | --- | | --- |
| Epoch^3^ X 2-D vs. 3-D, *β_70_* | | --- | | --- |
| Gender, *β_71_* | | --- | | --- |
| RSA | |  | |  |
| 2-D vs. 3-D, *β_40_* | | -0.09 | | 0.07 |
| Gender, *β_41_* | | 0.18 | | 0.15 |
| Epoch X 2-D vs. 3-D, *β_50_* | | 0.01 | | 0.03 |
| Gender, *β_51_* | | -0.09 | | 0.05 |
| Epoch^2^ X 2-D vs. 3-D, *β_60_* | | --- | | --- |
| Gender, *β_61_* | | --- | | --- |
| Epoch^3^ X 2-D vs. 3-D, *β_70_* | | --- | | --- |
| Gender, *β_71_* | | --- | | --- |
| Tonic period | |  | |  |
| 2-D vs. 3-D, *β_40_* | | -0.44 | | 0.58 |
| Gender, *β_41_* | | 0.08 | | 1.16 |
| Epoch X 2-D vs. 3-D, *β_50_* | | 1.01 | | 0.58 |
| Gender, *β_51_* | | 1.15 | | 1.16 |
| Epoch^2^ X 2-D vs. 3-D, *β_60_* | | -0.30 | | 0.18 |
| Gender, *β_61_* | | -0.44 | | 0.36 |
| Epoch^3^ X 2-D vs. 3-D, *β_70_* | | --- | | --- |
| Gender, *β_71_* | | --- | | --- |
|  | My Bloody Valentine | |  | |
| EDA | |  | |  |
| 2-D vs. 3-D, *β_40_* | | 0.08 | | 0.19 |
| Gender, *β_41_* | | 0.27 | | 0.39 |
| Epoch X 2-D vs. 3-D, *β_50_* | | -0.17 | | 0.13 |
| Gender, *β_51_* | | -0.36 | | 0.27 |
| Epoch^2^ X 2-D vs. 3-D, *β_60_* | | 0.03 | | 0.03 |
| Gender, *β_61_* | | 0.11 | | 0.06 |
| Epoch^3^ X 2-D vs. 3-D, *β_70_* | | --- | | --- |
| Gender, *β_71_* | | --- | | --- |
| Heart rate | |  | |  |
| 2-D vs. 3-D, *β_40_* | | -0.16 | | 0.50 |
| Gender, *β_41_* | | 0.28 | | 1.00 |
| Epoch X 2-D vs. 3-D, *β_50_* | | 0.23 | | 0.18 |
| Gender, *β_51_* | | -0.29 | | 0.36 |
| Epoch^2^ X 2-D vs. 3-D, *β_60_* | | --- | | --- |
| Gender, *β_61_* | | --- | | --- |
| Epoch^3^ X 2-D vs. 3-D, *β_70_* | | --- | | --- |
| Gender, *β_71_* | | --- | | --- |
| PEP | |  | |  |
| 2-D vs. 3-D, *β_40_* | | 0.27 | | 0.47 |
| Gender, *β_41_* | | 0.12 | | 0.94 |
| Epoch X 2-D vs. 3-D, *β_50_* | | -0.12 | | 0.14 |
| Gender, *β_51_* | | -0.19 | | 0.28 |
| Epoch^2^ X 2-D vs. 3-D, *β_60_* | | --- | | --- |
| Gender, *β_61_* | | --- | | --- |
| Epoch^3^ X 2-D vs. 3-D, *β_70_* | | --- | | --- |
| Gender, *β_71_* | | --- | | --- |
| RSA | |  | |  |
| 2-D vs. 3-D, *β_40_* | | 0.13 | | 0.09 |
| Gender, *β_41_* | | -0.06 | | 0.18 |
| Epoch X 2-D vs. 3-D, *β_50_* | | -0.01 | | 0.07 |
| Gender, *β_51_* | | 0.16 | | 0.14 |
| Epoch^2^ X 2-D vs. 3-D, *β_60_* | | -0.01 | | 0.02 |
| Gender, *β_61_* | | -0.02 | | 0.03 |
| Epoch^3^ X 2-D vs. 3-D, *β_70_* | | --- | | --- |
| Gender, *β_71_* | | --- | | --- |
| Tonic period | |  | |  |
| 2-D vs. 3-D, *β_40_* | | -0.07 | | 0.62 |
| Gender, *β_41_* | | -.91 | | 1.21 |
| Epoch X 2-D vs. 3-D, *β_50_* | | -0.19 | | 0.39 |
| Gender, *β_51_* | | 0.63 | | 0.79 |
| Epoch^2^ X 2-D vs. 3-D, *β_60_* | | 0.06 | | 0.09 |
| Gender, *β_61_* | | -0.28 | | 0.18 |
| Epoch^3^ X 2-D vs. 3-D, *β_70_* | | --- | | --- |
| Gender, *β_71_* | | --- | | --- |
|  | Polar Express | |  | |
| EDA | |  | |  |
| 2-D vs. 3-D, *β_40_* | | 0.58^**^ | | 0.19 |
| Gender, *β_41_* | | -0.26 | | 0.38 |
| Epoch X 2-D vs. 3-D, *β_50_* | | -0.40^***^ | | 0.12 |
| Gender, *β_51_* | | 0.19 | | 0.25 |
| Epoch^2^ X 2-D vs. 3-D, *β_60_* | | 0.08^**^ | | 0.03 |
| Gender, *β_61_* | | -0.07 | | 0.06 |
| Epoch^3^ X 2-D vs. 3-D, *β_70_* | | --- | | --- |
| Gender, *β_71_* | | --- | | --- |
| Heart rate | |  | |  |
| 2-D vs. 3-D, *β_40_* | | 0.34 | | 0.34 |
| Gender, *β_41_* | | -0.70 | | 0.69 |
| Epoch X 2-D vs. 3-D, *β_50_* | | 0.22 | | 0.53 |
| Gender, *β_51_* | | -0.93 | | 1.06 |
| Epoch^2^ X 2-D vs. 3-D, *β_60_* | | -0.25 | | 0.33 |
| Gender, *β_61_* | | 0.34 | | 0.66 |
| Epoch^3^ X 2-D vs. 3-D, *β_70_* | | 0.05 | | 0.05 |
| Gender, *β_71_* | | -0.03 | | 0.11 |
| PEP | |  | |  |
| 2-D vs. 3-D, *β_40_* | | -1.02 | | 0.86 |
| Gender, *β_41_* | | 0.26 | | 1.72 |
| Epoch X 2-D vs. 3-D, *β_50_* | | 0.59 | | 0.55 |
| Gender, *β_51_* | | -0.80 | | 1.10 |
| Epoch^2^ X 2-D vs. 3-D, *β_60_* | | -0.06 | | 0.09 |
| Gender, *β_61_* | | 0.12 | | 0.18 |
| Epoch^3^ X 2-D vs. 3-D, *β_70_* | | --- | | --- |
| Gender, *β_71_* | | --- | | --- |
| RSA | |  | |  |
| 2-D vs. 3-D, *β_40_* | | 0.05 | | 0.11 |
| Gender, *β_41_* | | -0.21 | | 0.22 |
| Epoch X 2-D vs. 3-D, *β_50_* | | 0.02 | | 0.08 |
| Gender, *β_51_* | | 0.09 | | 0.16 |
| Epoch^2^ X 2-D vs. 3-D, *β_60_* | | 0.00 | | 0.01 |
| Gender, *β_61_* | | -0.02 | | 0.03 |
| Epoch^3^ X 2-D vs. 3-D, *β_70_* | | --- | | --- |
| Gender, *β_71_* | | --- | | --- |
| Tonic period | |  | |  |
| 2-D vs. 3-D, *β_40_* | | -0.34 | | 0.52 |
| Gender, *β_41_* | | 2.14^*^ | | 0.14 |
| Epoch X 2-D vs. 3-D, *β_50_* | | -0.67 | | 0.40 |
| Gender, *β_51_* | | -0.03 | | 0.79 |
| Epoch^2^ X 2-D vs. 3-D, *β_60_* | | 0.18 | | 0.09 |
| Gender, *β_61_* | | 0.02 | | 0.19 |
| Epoch^3^ X 2-D vs. 3-D, *β_70_* | | --- | | --- |
| Gender, *β_71_* | | --- | | --- |
|  | Tangled | |  | |
| EDA | |  | |  |
| 2-D vs. 3-D, *β_40_* | | 0.35^*^ | | 0.18 |
| Gender, *β_41_* | | -0.01 | | 0.35 |
| Epoch X 2-D vs. 3-D, *β_50_* | | -0.32 | | 0.28 |
| Gender, *β_51_* | | -0.10 | | 0.53 |
| Epoch^2^ X 2-D vs. 3-D, *β_60_* | | 0.20 | | 0.16 |
| Gender, *β_61_* | | 0.08 | | 0.32 |
| Epoch^3^ X 2-D vs. 3-D, *β_70_* | | -0.04 | | 0.03 |
| Gender, *β_71_* | | -0.01 | | 0.05 |
| Heart rate | |  | |  |
| 2-D vs. 3-D, *β_40_* | | 0.43 | | 0.45 |
| Gender, *β_41_* | | 0.13 | | 0.90 |
| Epoch X 2-D vs. 3-D, *β_50_* | | -0.40 | | 0.53 |
| Gender, *β_51_* | | 0.61 | | 1.05 |
| Epoch^2^ X 2-D vs. 3-D, *β_60_* | | 0.13 | | 0.34 |
| Gender, *β_61_* | | -0.34 | | 0.68 |
| Epoch^3^ X 2-D vs. 3-D, *β_70_* | | -0.01 | | 0.06 |
| Gender, *β_71_* | | 0.06 | | 0.11 |
| PEP | |  | |  |
| 2-D vs. 3-D, *β_40_* | | -1.26 | | 0.66 |
| Gender, *β_41_* | | -0.32 | | 1.32 |
| Epoch X 2-D vs. 3-D, *β_50_* | | 0.88 | | 0.74 |
| Gender, *β_51_* | | -0.21 | | 1.47 |
| Epoch^2^ X 2-D vs. 3-D, *β_60_* | | -0.48 | | 0.46 |
| Gender, *β_61_* | | -0.26 | | 0.93 |
| Epoch^3^ X 2-D vs. 3-D, *β_70_* | | 0.08 | | 0.08 |
| Gender, *β_71_* | | 0.07 | | 0.15 |
| RSA | |  | |  |
| 2-D vs. 3-D, *β_40_* | | 0.12 | | 0.08 |
| Gender, *β_41_* | | -0.04 | | 0.16 |
| Epoch X 2-D vs. 3-D, *β_50_* | | -0.04 | | 0.07 |
| Gender, *β_51_* | | -0.02 | | 0.13 |
| Epoch^2^ X 2-D vs. 3-D, *β_60_* | | 0.011 | | 0.02 |
| Gender, *β_61_* | | 0.01 | | 0.03 |
| Epoch^3^ X 2-D vs. 3-D, *β_70_* | | --- | | --- |
| Gender, *β_71_* | | --- | | --- |
| Tonic period | |  | |  |
| 2-D vs. 3-D, *β_40_* | | 0.86 | | 0.53 |
| Gender, *β_41_* | | -0.50 | | 1.06 |
| Epoch X 2-D vs. 3-D, *β_50_* | | -0.74 | | 0.92 |
| Gender, *β_51_* | | 0.29 | | 1.85 |
| Epoch^2^ X 2-D vs. 3-D, *β_60_* | | -0.05 | | 0.58 |
| Gender, *β_61_* | | -0.55 | | 1.16 |
| Epoch^3^ X 2-D vs. 3-D, *β_70_* | | 0.07 | | 0.10 |
| Gender, *β_71_* | | 0.10 | | 0.19 |

*Note:* Robust standard errors are reported

^*^ p < .05, ^**^ p < .01, ^***^ p < .001
