# Supplementary material for: Testing the Effectiveness of 3D Film for Laboratory-Based Studies of Emotion
Source: PLoS One. 2014 Aug 29;9(8):e105554. doi: 10.1371/journal.pone.0105554 (PMC4149373; doi:10.1371/journal.pone.0105554)
Supplement: Table S3 — Results of Analyses Testing whether Individual Differences Moderate the 3D Response. Robust standard errors are reported. * p<.05, ** p<.01, *** p<.001. (DOCX) [file pone.0105554.s003.docx]

Table S3

*Results of analyses testing whether individual differences moderate the 3D response*

| RSA DERS | Despicable Me | | | | My Bloody Valentine | | | Polar Express | | | Tangled | |
| --- | --- | --- | --- | --- | --- | --- | --- | --- | --- | --- | --- | --- |
|  | B | | | SE | B | | SE | B | | SE | B | SE |
| Intercept, β_00_ | 0.06 | | | 0.07 | 0.00 | | 0.09 | -0.25^**^ | | 0.09 | -0.19^*^ | 0.08 |
| Gender, β_01_ | 0.06 | | | 0.14 | -0.11 | | 0.17 | -0.06 | | 0.18 | 0.08 | 0.15 |
| DERS, β_02_ | 0.00 | | | 0.00 | 0.00 | | 0.01 | 0.01^**^ | | 0.00 | 0.00 | 0.01 |
| Epoch, β_10_ | -0.07^***^ | | | 0.02 | -0.34^***^ | | 0.06 | 0.25^***^ | | 0.06 | -0.19^***^ | 0.05 |
| Gender, β_11_ | 0.06 | | | 0.05 | 0.01 | | 0.12 | -0.15 | | 0.12 | 0.11 | 0.11 |
| DERS, β_12_ | 0.00 | | | 0.00 | 0.00 | | 0.00 | -0.01^***^ | | 0.00 | 0.00 | 0.00 |
| Epoch^2^, β_20_ | --- | | | --- | 0.04^***^ | | 0.01 | -0.06^***^ | | 0.01 | 0.05^***^ | 0.01 |
| Gender, β_21_ | --- | | | --- | -0.01 | | 0.03 | 0.02 | | 0.02 | -0.03 | 0.03 |
| DERS, β_22_ | --- | | | --- | 0.00 | | 0.00 | 0.00^***^ | | 0.00 | 0.00 | 0.00 |
| Epoch^3^, β_30_ | --- | | | --- | --- | | --- | --- | | --- | --- | --- |
| Gender, β_31_ | --- | | | --- | --- | | --- | --- | | --- | --- | --- |
| DERS, β_32_ | --- | | | --- | --- | | --- | --- | | --- | --- | --- |
| 2D vs. 3D, β_40_ | -0.07 | | | 0.08 | 0.13 | | 0.09 | 0.01 | | 0.11 | 0.12 | 0.08 |
| Gender, β_41_ | 0.17 | | | 0.15 | -0.06 | | 0.18 | -0.23 | | 0.23 | -0.03 | 0.17 |
| DERS, β_42_ | 0.01 | | | 0.00 | 0.00 | | 0.01 | 0.00 | | 0.00 | 0.00 | 0.00 |
| Epoch X 2D vs. 3D, β_50_ | 0.02 | | | 0.03 | -0.03 | | 0.06 | 0.03 | | 0.09 | -0.04 | 0.06 |
| Gender, β_51_ | -0.10 | | | 0.05 | 0.16 | | 0.14 | 0.14 | | 0.17 | -0.02 | 0.14 |
| DERS, β_52_ | 0.00 | | | 0.00 | 0.00 | | 0.00 | 0.00 | | 0.00 | 0.01^*^ | 0.00 |
| Epoch^2^ X 2D vs. 3D, β_60_ | --- | | | --- | 0.00 | | 0.01 | 0.00 | | 0.01 | 0.01 | 0.01 |
| Gender, β_61_ | --- | | | --- | -0.02 | | 0.03 | -0.03 | | 0.03 | 0.01 | 0.03 |
| DERS, β_62_ | --- | | | --- | 0.00 | | 0.00 | 0.00 | | 0.00 | 0.00^*^ | 0.00 |
| Epoch^3^ X 2D vs. 3D, β_70_ | --- | | | --- | --- | | --- | --- | | --- | --- | --- |
| Gender, β_71_ | --- | | | --- | --- | | --- | --- | | --- | --- | --- |
| DERS, β_72_ | --- | | | --- | --- | | --- | --- | | --- | --- | --- |
| PEP Novelty Seeking | Despicable Me | | | | My Bloody Valentine | | | Polar Express | | | Tangled | |
|  | B | | | SE | B | | SE | B | | SE | B | SE |
| Intercept, β_00_ | -1.17^**^ | | | 0.38 | -1.74^***^ | | 0.47 | -1.01 | | 0.72 | 0.57 | 0.59 |
| Gender, β_01_ | -1.17 | | | 0.77 | 0.64 | | 0.94 | 0.22 | | 1.45 | -0.78 | 1.17 |
| NS, β_02_ | 0.01 | | | 0.04 | 0.09 | | 0.06 | -0.04 | | 0.07 | -0.01 | 0.07 |
| Epoch, β_10_ | 0.56^***^ | | | 0.12 | -0.79^***^ | | 0.14 | 0.42 | | 0.58 | 0.87 | 0.72 |
| Gender, β_11_ | 0.14 | | | 0.25 | 0.37 | | 0.27 | 1.19 | | 1.15 | 1.66 | 1.43 |
| NS, β_12_ | 0.00 | | | 0.01 | 0.01 | | 0.01 | 0.06 | | 0.05 | 0.01 | 0.08 |
| Epoch^2^, β_20_ | --- | | | --- | --- | | --- | -0.04 | | 0.10 | -0.56 | 0.42 |
| Gender, β_21_ | --- | | | --- | --- | | --- | -0.22 | | 0.19 | -1.11 | 0.84 |
| NS, β_22_ | --- | | | --- | --- | | --- | -0.01 | | 0.01 | -0.01 | 0.05 |
| Epoch^3^, β_30_ | --- | | | --- | --- | | --- | --- | | --- | 0.09 | 0.12 |
| Gender, β_31_ | --- | | | --- | --- | | --- | --- | | --- | 0.17 | 0.25 |
| NS, β_32_ | --- | | | --- | --- | | --- | --- | | --- | 0.00 | 0.01 |
| 2D vs. 3D, β_40_ | 0.31 | | | 0.45 | 0.24 | | 0.46 | -0.97 | | 0.95 | -1.24 | 0.68 |
| Gender, β_41_ | 0.75 | | | 0.94 | -0.06 | | 0.96 | 0.31 | | 1.90 | -0.35 | 1.32 |
| NS, β_42_ | -0.04 | | | 0.05 | 0.00 | | 0.05 | -0.05 | | 0.08 | 0.03 | 0.07 |
| Epoch X 2D vs. 3D, β_50_ | 0.03 | | | 0.16 | -0.12 | | 0.14 | 0.76 | | 0.64 | 0.96 | 0.73 |
| Gender, β_51_ | -0.42 | | | 0.32 | -0.20 | | 0.28 | -1.02 | | 1.25 | -0.31 | 1.46 |
| NS, β_52_ | -0.02 | | | 0.01 | -0.02 | | 0.01 | 0.02 | | 0.06 | 0.06 | 0.09 |
| Epoch^2^ X 2D vs. 3D, β_60_ | --- | | | --- | --- | | --- | -0.10 | | 0.10 | -0.50 | 0.85 |
| Gender, β_61_ | --- | | | --- | --- | | --- | -0.17 | | 0.20 | -0.21 | 1.69 |
| NS, β_62_ | --- | | | --- | --- | | --- | 0.00 | | 0.01 | -0.04 | 0.10 |
| Epoch^3^ X 2D vs. 3D, β_70_ | --- | | | --- | --- | | --- | --- | | --- | 0.08 | 0.14 |
| Gender, β_71_ | --- | | | --- | --- | | --- | --- | | --- | 0.07 | 0.28 |
| NS, β_72_ | --- | | | --- | --- | | --- | --- | | --- | 0.01 | 0.02 |
| EDA Trait Anxiety | | Despicable Me | | | My Bloody Valentine | | | Polar Express | | | Tangled | |
|  | | B | SE | | B | SE | | B | SE | | B | SE |
| Intercept, β_00_ | | 1.13^***^ | 0.15 | | 2.36^***^ | 0.23 | | 2.21^***^ | 0.17 | | 1.86^***^ | 0.18 |
| Gender, β_01_ | | 0.25 | 0.30 | | -0.94^*^ | 0.45 | | 0.03 | 0.34 | | 0.19 | 0.36 |
| Trait Anxiety, β_02_ | | -0.01 | 0.02 | | -0.02 | 0.03 | | -0.02 | 0.01 | | 0.02 | 0.02 |
| Epoch, β_10_ | | -0.55^***^ | 0.15 | | -0.11 | 0.17 | | -1.53^***^ | 0.14 | | -2.26^***^ | 0.25 |
| Gender, β_11_ | | 0.35 | 0.30 | | 1.35^***^ | 0.32 | | 0.27 | 0.28 | | -0.02 | 0.51 |
| Trait Anxiety, β_12_ | | 0.01 | 0.01 | | -0.01 | 0.02 | | 0.01 | 0.01 | | 0.01 | 0.03 |
| Epoch^2^, β_20_ | | 0.14^**^ | 0.05 | | 0.05 | 0.03 | | 0.28^***^ | 0.03 | | 1.29^***^ | 0.15 |
| Gender, β_21_ | | -0.12 | 0.09 | | -0.30^***^ | 0.07 | | -0.03 | 0.06 | | 0.04 | 0.31 |
| Trait Anxiety, β_22_ | | 0.00 | 0.00 | | 0.00 | 0.00 | | 0.00 | 0.00 | | -0.01 | 0.02 |
| Epoch^3^, β_30_ | | --- | --- | | --- | --- | | --- | --- | | -0.20^***^ | 0.02 |
| Gender, β_31_ | | --- | --- | | --- | --- | | --- | --- | | -0.01 | 0.05 |
| Trait Anxiety, β_32_ | | --- | --- | | --- | --- | | --- | --- | | 0.00 | 0.00 |
| 2D vs. 3D, β_40_ | | -0.08 | 0.16 | | 0.15 | 0.21 | | 0.56^**^ | 0.19 | | 0.35^*^ | 0.17 |
| Gender, β_41_ | | 0.20 | 0.31 | | 0.16 | 0.42 | | -0.21 | 0.38 | | -0.05 | 0.34 |
| Trait Anxiety, β_42_ | | -0.02 | 0.01 | | 0.02 | 0.02 | | 0.01 | 0.01 | | -0.01 | 0.02 |
| Epoch X 2D vs. 3D, β_50_ | | -0.29 | 0.16 | | -0.20 | 0.14 | | -0.41^***^ | 0.12 | | -0.33 | 0.27 |
| Gender, β_51_ | | -0.26 | 0.33 | | -0.48 | 0.29 | | 0.21 | 0.24 | | -0.12 | 0.54 |
| Trait Anxiety, β_52_ | | 0.01 | 0.02 | | -0.01 | 0.02 | | 0.00 | 0.01 | | 0.01 | 0.02 |
| Epoch^2^ X 2D vs. 3D, β_60_ | | 0.07 | 0.05 | | 0.04 | 0.03 | | 0.08^***^ | 0.03 | | 0.21 | 0.16 |
| Gender, β_61_ | | 0.11 | 0.10 | | 0.13^*^ | 0.06 | | -0.07 | 0.05 | | 0.10 | 0.33 |
| Trait Anxiety, β_62_ | | 0.00 | 0.01 | | 0.00 | 0.00 | | 0.00 | 0.00 | | -0.01 | 0.01 |
| Epoch^3^ X 2D vs. 3D, β_70_ | | --- | --- | | --- | --- | | --- | --- | | -0.04 | 0.03 |
| Gender, β_71_ | | --- | --- | | --- | --- | | --- | --- | | 0.02 | 0.05 |
| Trait Anxiety, β_72_ | | --- | --- | | --- | --- | | --- | --- | | 0.00 | 0.00 |

*Note:* Robust standard errors are reported

^*^ p < .05, ^**^ p < .01, ^***^ p < .001
